# Signal strength and signal duration define two distinct aspects of JNK-regulated axon stability

**DOI:** 10.1016/j.ydbio.2009.12.016

**Published:** 2010-03-01

**Authors:** Andrew Rallis, Coralie Moore, Julian Ng

**Affiliations:** MRC Centre for Developmental Neurobiology, New Hunt's House, Guy's Campus, King's College, London SE1 1UL, UK

**Keywords:** Jun N-terminal kinase, Fos, Jun, AP-1, Axonal morphogenesis, Neurodegeneration, Neural development, *Drosophila*

## Abstract

Signaling proteins often control multiple aspects of cell morphogenesis. Yet the mechanisms that govern their pleiotropic behavior are often unclear. Here we show activity levels and timing mechanisms determine distinct aspects of Jun N-terminal kinase (JNK) pathway dependent axonal morphogenesis in *Drosophila* mushroom body (MB) neurons. In the complete absence of *Drosophila* JNK (Basket), MB axons fail to stabilize, leading to their subsequent degeneration. However, with a partial loss of Basket (Bsk), or of one of the upstream JNK kinases, Hemipterous or Mkk4, these axons overextend. This suggests that Bsk activity prevents axons from destabilizing, resulting in degeneration and overextension beyond their terminal targets. These distinct phenotypes require different threshold activities involving the convergent action of two distinct JNK kinases. We show that sustained Bsk signals are essential throughout development and act additively but are dispensable at adulthood. We also suggest that graded Bsk inputs are translated into AP-1 transcriptional outputs consisting of Fos and Jun proteins.

## Introduction

To reach maturity, developing neurons undergo many morphogenetic changes including axon and dendrite formation/polarity, neurite extension, guidance, branching and synaptogenesis. The mechanisms that underlie these distinct steps are not well understood. This results partly from the observation that, although many molecules are involved, they exhibit pleiotropy, controlling several aspects of neuronal morphogenesis. The MAPK family of signaling proteins epitomizes such pleiotropic factors. They are present throughout eukaryotes and control many cellular responses, such as proliferation, differentiation, stress and apoptotic control (Weston and Davis, 2002). MAPKs (Erk, JNK or p38 members) are activated through phosphorylation by upstream kinases, which are themselves regulated by other protein kinases. Several studies show the Jun N-terminal kinase (JNK) pathway is involved in axon formation/polarization, extension, synaptic plasticity and dendrite development (Oliva et al., 2006; Rosso et al., 2005; Sanyal et al., 2002; Srahna et al., 2006). Many non-neuronal models have been used to explain how JNKs regulate multiple aspects of cell regulation. For example, one proposal is that the core-signaling component is linked to distinct specialized complexes. JNKs are regulated by distinct upstream kinases through interactions with scaffold proteins (Weston and Davis, 2002). These link JNK responses to particular stimuli, such as morphogenetic, stress or apoptotic regulation. However, timing mechanisms can also play a role. From chemical genetic paradigms using JNK mutant mice, transient or prolonged JNK inactivation can affect distinct JNK-dependent immune responses (Ventura et al., 2006). Another possibility is that signaling molecules have context-dependent roles in different cell types. Thus, while in mammalian hippocampal cells, JNKs are involved in dendritogenesis (Rosso et al., 2005) and axonal polarity/formation (Oliva et al., 2006), in *Drosophila* dorsal cluster (DC) neurons, JNK is involved in axon extension (Srahna et al., 2006), and at the *Drosophila* neuromuscular junction (NMJ), JNK regulates synaptic plasticity and growth (Collins et al., 2006; Sanyal et al., 2002).

In *Drosophila*, the JNK signaling network consists of one JNK, Basket (Bsk), which is regulated by two JNK kinases (JNKKs), Hemipterous (Hep) and MAP kinase kinase 4 (MKK4) (Glise et al., 1995; Han et al., 1998; Riesgo-Escovar and Hafen, 1997b). Six JNKK kinases (JNKKKs) exist, which control the JNKKs (Stronach, 2005). Further upstream, a single JNKKKK, Misshapen (Msn), regulates the JNKKKs (Su et al., 1998). Like in many other model systems studied, Bsk responses in *Drosophila* are varied. While first shown to control epithelial morphogenesis during embryonic dorsal closure, Bsk also contributes to imaginal disk development, apoptotic regulation, wound healing, tissue regeneration, tissue homeostasis and innate immunity (Agnes et al., 1999; Bosch et al., 2005; Delaney et al., 2006; Galko and Krasnow, 2004; Mattila et al., 2005; Noselli, 1998; Pastor-Pareja et al., 2004; Ramet et al., 2002; Ryoo et al., 2004; Sluss et al., 1996; Vidal et al., 2001; Zeitlinger et al., 1997). Additionally, Bsk signals can prolong lifespan and protect against oxidative stress in flies (Wang et al., 2003).

In many of these responses, Bsk targets the phosphorylation of the Activator protein-1 (AP-1) complex, composed of the transcription factors Fos and Jun (Ciapponi et al., 2001; Kockel et al., 1997). In *Drosophila*, these act either as heterodimers or as Fos homodimers (Pearson et al., 2009; Perkins et al., 1990). In many studies, distinct JNKKKs are thought to represent stimulus-specific regulators and the core components are represented by the Hep→Bsk→AP-1 response (Stronach, 2005). The in vivo role of MKK4 has not been previously described.

Here we show that *Drosophila* JNK controls two distinct axonal phenotypes in mushroom body (MB) neurons. Depending on the level of Bsk inactivation, this results in a bias towards axon degeneration or overextension. Based on these phenotypes, we propose that Bsk controls axon stabilization via two mechanisms, to prevent axons from degenerating and from overextending beyond the postsynaptic target. These mechanisms require different threshold activity levels, involving the upstream JNKKs, Hep and Mkk4. We show that sustained Bsk activity is required throughout development to maintain axonal stability. These phenotypes are mediated through AP-1, which shows a similar graded response to these axonal phenotypes.

## Materials and methods

### Drosophila strains

*bsk^147e^*and *hep* mutant strains (*hep^R39^*and *hep*^*75*^) are null mutations, as previously described (Glise et al., 1995; Sluss et al., 1996). The *bsk^H15^* allele (Berger et al., 2008) encodes a missense mutation (I212F-PB isoform) within the kinase domain, which is likely to result in a hypomorphic allele (see quantifications in Fig. 6I) The *Mkk4*^*e01458*^ allele is derived from a *PiggyBac* insertion (Thibault et al., 2004). *Mkk4^e01458^*complemented the lethality associated with chromosomal deficiencies *Df(3R)Dhod15*, *Df(3R)BSC195* and *Df(3R)BSC197* but failed to complement *Df(3R)p13* and *Df(3R)Exel6149* (Flybase). Ubiquitous expression of Mkk4 (*tub-GAL4>MKK4YFP*) fully rescued the lethality associated with *Mkk4^e01458^/Df(3R)p13* transheterozygotes, confirming that the insertion disrupts the *Mkk4* locus.

The following additional strains were also used in our study; *UAS-Bskmyc, UAS-Mkk4Venus* (this study); *UAS-Bsk RNAi, UAS-Mkk4 RNAi* (VDRC lines 34138, 34139 and 26928), *UAS-Dcr2* (Dietzl et al., 2007); *UAS-Bsk DN*, *UAS-Hep.B*^*2*^, *UAS-Hep.CA*^*4*^ and *GAL80*^*ts7*^ (Bloomington Drosophila Stock Center); *Jun*^*2*^, *Jun*^*1*^ (Kockel et al., 1997); *UAS-Jbz, UAS-Fbz,* (Eresh et al., 1997); *UAS-kay RNAi, UAS-hep RNAi* (NIG-Fly, Mishima; lines 15507R-4 and 2190R-1); *kay^1^*(Riesgo-Escovar and Hafen, 1997a; Zeitlinger et al., 1997); *kay*^*ED6315*^ (Weber et al., 2008). These *kay* alleles are not true nulls, as they do not disrupt all Kay isoforms (Giesen et al., 2003; Weber et al., 2008). *Drosophila* strains for MARCM analysis have previously been described (Lee and Luo, 1999) and mutant strains were generated by standard recombination techniques.

### Molecular biology

To generate expression vectors *pUAST-Bskmyc* and *pUAST-Mkk4YFP*, Bsk and Mkk4 cDNAs (a gift from D. Bohmann and BDGP clone RE70055, respectively) were cloned into a pENTR vector (Invitrogen) by PCR and TOPO^®^ cloning. The resulting pENTR-*Bsk* and pENTR-*Mkk4* clones were ligated to the destination vectors (pTWM or pTWV, respectively; T. Murphy, Carnegie *Drosophila* Gateway^®^ vectors) using the Gateway^®^ system (Invitrogen). *pUAST-BskMyc^T181A, Y183F^* was constructed by site directed mutagenesis (Quikchange^®^, Stratagene) and cloning into the pTWM vector, as above. Germline transformations were performed commercially (Aktogen, Cambridge, UK).

### Generation of MARCM clones, UAS-Gal4, RNAi and TARGET expression analysis

Homozygous mutant clones that are positively labeled were generated using the MARCM method. MB neuroblast and single-cell clones were generated as previously described (Wu and Luo, 2006b). A single neuroblast clone can give rise to ∼600 Kenyon cells (Ito et al., 1997). Neurons were visualized using the OK107-Gal4 driver expressing mCD8GFP. The Gal4-OK107 driver was also used in misexpression studies, along with one copy of UAS-CD8GFP. Flies were reared at 25 °C, unless otherwise stated such as in RNAi and TARGET experiments. For ‘high’ level of RNAi knockdown, flies were cultured at 29 °C in the presence of ectopic Dicer (Dcr2), to increase the level of UAS expression and RNAi efficiency (Dietzl et al., 2007). For ‘medium’ RNAi activity, flies were raised at 29 °C without ectopic Dcr2. We found that even at low levels of RNAi expression (18 °C), Dcr2 expression can significantly enhance RNAi phenotypes. Ectopic expression of Dcr2 alone does not disrupt MB axon projections (data not shown). Flies were dissected within 3–7 days post-eclosion. For the TARGET protocol, flies were grown at 18 °C and UAS-GAL4 expression was induced by transferring to 29 °C at the indicated stages. In the ‘reverse’ protocol, flies were grown at 29 °C and shifted to 18 °C. These flies were maintained in the shifted temperatures and analyzed at 3 days post-eclosion, unless indicated otherwise.

### Immunohistochemistry

Fly brains were dissected at various stages and stained as previously described (Wu and Luo, 2006a). For MARCM neuroblast and single-cell clones, axon projections were visualized using anti-FasII (1:5) and anti-mCD8, 1:200. The following additional antibodies were used: anti-JNK1 (Santa Cruz Biotechnology, sc-571, 1:250), anti-phospho JNK (Cell Signalling, no. 9255, 1:250), anti-Myc (Santa Cruz Biotechnology, clone 9E10; 1:100 or Cell Signalling, no. 2272; 1:200), anti-GFP (Molecular Probes, A11122, 1:100 or Roche, 11814460001, 1:200), anti-MKK4 (1:50) and anti-Hep (1:100). Anti-Hep and anti-Mkk4 antibodies were generated commercially using rabbit and guinea pig hosts, respectively, using a DXP protocol (Eurogentec, Seraing, Belgium). The following peptide sequences were used as immunogens (QSLEAKLQAQNESHDC and CLRANGDPTLQRLPNS for Hep; MAERPKNLFATGSSRC and CKDGITQFTANQQAES for Mkk4). Stained brains were imaged by confocal microscopy (using Zeiss 510 and processed using Zeiss LSM and Adobe photoshop software).

### S2 cell culture, expression and Western blotting analysis

*Drosophila* S2 cells were maintained at 25 °C in Schneider's media, supplemented with 10% fetal bovine serum. S2 transfections were performed using the pMT-GAL4 binary system (Klueg et al., 2002). Briefly, 2 × 10^6^ cells (50% confluent) were plated in 2 ml of medium 24 h prior to the transfection. 2 μg of pUAST and 2 μg of pMT-GAL4 plasmids were then added per well (90% cell confluent stage), along with 30 μl of Cellfectin (Invitrogen) in 2 ml of antibiotic-free, Schneider's Media. The media were removed 3 h post-transfection and replaced with media supplemented with 10% fetal bovine serum, 1% streptomycin (Gibco BRL). Twelve hours later, the media were replaced with media containing 1% CuSO_4_ to induce protein expression. S2 cells were subsequently harvested 24 h later. Cells were harvested by centrifugation at 1000  *g* for 5 min and lysed on ice for 30 min in RIPA buffer [10 mM Tris (pH 7.4), 10 mM NaH_2_PO_4_, 150 mM NaCl, 1% Triton X-100], supplemented with protease and phosphatase inhibitors (Halt^®^ protease inhibitor from Pierce, 10 mM NaF and 1 mM Na_3_VO_4_). Lysates were spun for 10 min at 21,000 *g*. The supernatant was added to reducing sample loading buffer (2) in equal quantity. Reduced protein samples were run on 10% polyacrylamide Tris–HCl gels and transferred to Invitrolon® membranes (Invitrogen), using standard methods (Bio-Rad). Immunoblots were probed with phospho-JNK (Cell Signalling, no. 9255, 1:1000) and JNK1 (Santa Cruz Biotechnology, sc-571, 1:4000), Hep (ab1956; 1:2000 and ab1957; 1:250) and Mkk4 antibodies (ab1954 and 1955; both at 1:5000). Additional antibodies used were GFP (Molecular Probes, A11122, 1:4000) and myc (Santa Cruz Biotechnology, clone 9E10, 1:4000) using standard protocols. Blots were developed with Pico-ECL chemiluminescence reagents (Pierce) and exposed to ECL hyperfilm (Amersham Biosciences).

## Results

### *Drosophila* JNK activity is detected in MB axons

**Fig. 1:**
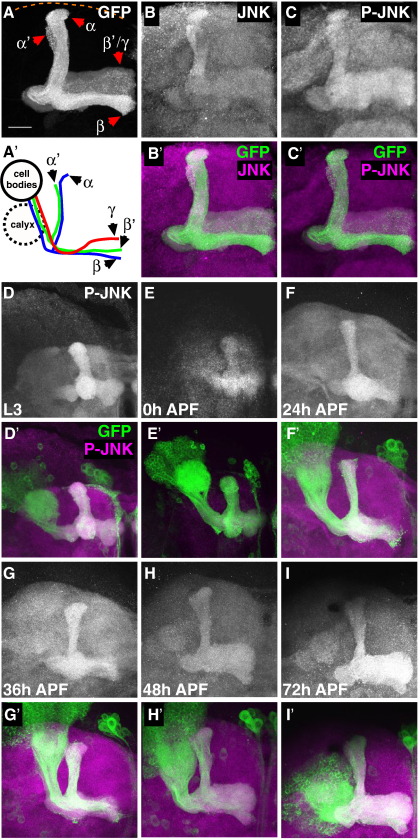
JNK is highly expressed in MB axons and dendrites. (A) MB axons labeled by CD8-GFP expression, using the OK107-Gal4 driver. These adult MB axonal projections are wild type. Axons terminate close to the midline (to the right of all images, unless indicated with a dashed white line), or close to the anterior dorsal cortex (dashed orange line). The different axon lobes (γ, α'β', αβ) are indicated, as previously (Lee et al., 1999). (A') A schematic of these MB neuron subtypes (γ, α'β', αβ) and the relative location of cell bodies, dendrite (‘calyx’) and axon projections. (B) The same brain immunostained with anti-JNK1, the overlap with the GFP marker (magenta in B') shows axonal localization. (C) The same brain also immunostained with anti-phospho JNK (P-JNK). (C') The overlap shows high JNK activity in MB axons. (D–I) P-JNK brain staining at various developmental stages, from wandering larvae L3 and pupae at different time-points after puparium formation (APF), indicated in hours (h). The additional panels (D'–I') show the corresponding overlap between P-JNK and the GFP labeled MB axons at these stages. Unless indicated otherwise, these and subsequent images are *z*-stack of serial confocal images taken at 1-μm thickness. In some images (such as in A), cell body sections have been omitted to clearly reveal axonal projections. Scale bar: 20 μm.

MB neurons (Kenyon cells) in the *Drosophila* brain were used as a model to analyze Bsk signaling in vivo (Ito et al., 1997; Kurusu et al., 2002; Lee et al., 1999). Adult MB neurons are composed of three distinct sets (γ, α'β' and αβ) with each set having distinct axonal projections (Lee et al., 1999)(Figs. 1A and A'). MB axons extend from posterior located cell bodies and lead to dorsal and medial projections in the anterior part of the brain. These projections terminate in the anterior dorsal cortex (α' and α), or close to the midline (γ, β' and β). Antibodies were used in whole-mount immunohistochemistry to determine Bsk activity in these neurons. Using a human JNK1 antibody that cross-reacts with the *Drosophila* protein, Bsk was detected in MB axons (Fig. 1B). Using a phosphorylation-specific antibody that detects active forms of JNK, we found that Bsk was highly phosphorylated in adult MB axons (Fig. 1C). Low Bsk and p-Bsk signals were also present throughout the brain. Other notable structures that showed high Bsk/p-Bsk signals included the antennal lobe (AL) and ellipsoid body (eb) regions (Supplementary Fig. 1). As antibody controls, JNK and phospho-JNK immunoreactivity were not present when Bsk was lost or when its activity was inhibited in MB neurons (Supplementary Fig. 1). Analysis at different developmental stages showed that Bsk is active in axons throughout development, from wandering L3 larvae to adulthood (Fig. 1).

### Bsk loss results in axon degeneration

**Fig. 2:**
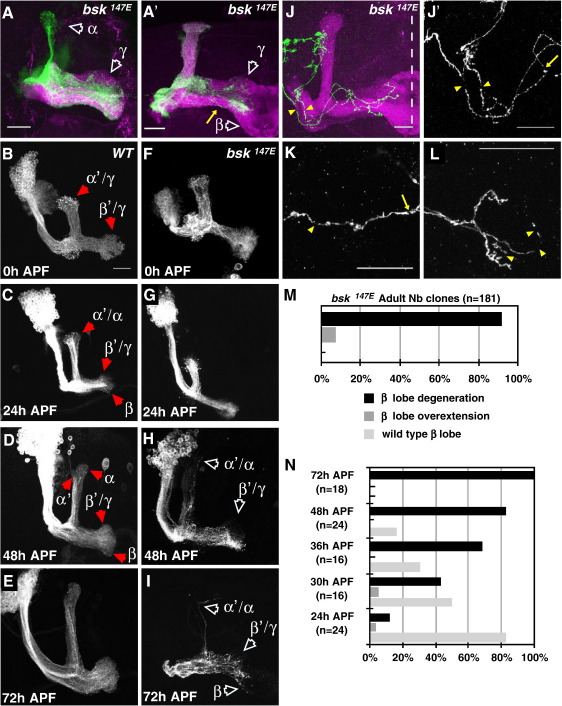
JNK loss results in axon destabilization. (A, A') Adult MB *bsk^147e^*neuroblast clones show axon thinning (yellow arrow) and termination defects (open white arrowheads). (B–I) Images of CD8-GFP labeled wild-type (B–E) and *bsk^147e^* (F–I) neuroblast clones analyzed at developmental stages: 0 h (B, F), 24 h (C, G), 48 h (D, H) and 72 h APF (E, I). At the onset of puparium formation (0 h APF), the majority of wild-type MB neurons consist of γ and α'β' neurons (B). A phase of neurogenesis occurs at this period and MB neuroblasts give rise to αβ neurons, which are visible at 24 h APF (C). As axon continue to grow, dorsal α and medial β lobes become more prominent. At 48 h APF, these lobes are similar to adult MB projections (D compare with Fig. 1A, respectively). Note *bsk* axonal defects at later stages of development (quantified in N). Axon defects were also more pronounced at later stages (compare H to I). The cell body section has been omitted from I. (J–L) Images of *bsk^147e^* single cell clones (γ-neurons) showing breaks (yellow arrowheads) along different regions of the axon. (J', K and L) Higher magnification of MB axons shows breaks and axon thinning in the proximal and mid-axonal shaft (J',K, respectively) and in the distal section (L). Scale bars: 20 μm. Unless indicated otherwise, CD8-GFP labeled neurons are shown in green or grayscale and Fas2 immunostaining (magenta) labels a subset (γ weakly and αβ strongly) of all MB axons. (M–N) Quantification of β-axon termination defects in adult MB *bsk^147e^* neuroblast clones (M), and at specific time points in development (N). *n*, number of neuroblast clones analyzed. While all MB axons displayed degeneration phenotypes to some extent, to simplify this study, we decided to focus on the medially projecting β-lobe. For *bsk* additional images and quantifications of other MB projections, see Supplementary Fig. 2.

The effect of Bsk inactivation was determined by generating MARCM neuroblast clones in MB neurons (see Methods). Analyzed at the adult stage, most *bsk*-null axons failed to reach the wild-type termination point (Figs. 2A, A', arrows). Many *bsk* clones also showed discontinuous thinning along the axon tracts, suggesting a possible axon loss (yellow arrow in Fig. 2A'). Interestingly, a minority of *bsk* axons displayed the converse phenotype with axon overextensions beyond their normal termination points (Supplementary Fig. 2A, B, open white arrows; quantifications in Fig. 2M and Supplementary Fig. 2I).

To determine whether this phenotype resulted from a failure in axon extension or stabilization, leading to subsequent degeneration, we analyzed *bsk* clones at different stages of development. The results showed the majority of *bsk* axons had normal, wild-type projections at early stages, but axonal defects characteristic of the adult stage were observed from 30 h after puparium formation (APF) onwards (Figs. 2F–I compared to wild type, Figs. 2B–E; quantified in Fig. 2N). These axon defects were often subtle at early to mid-pupal stages (data not shown; Fig. 2H) but becomes more acute in late pupae (Fig. 2I). At the adult stages, the entire axon lobe is often missing (Fig. 2A').

We analyzed this further by generating *bsk* single-cell clones. We found many fully extended axons showed thinning and breaks (Figs. 2J–L; compared to wild-type images in Supplementary Fig. 2C–E). This was not confined to axons, but also found in the main process close to the cell body, indicating an overall degeneration was taking place (Supplementary Fig. 2F, G compared to wild type, Fig. 2H). This was statistically significant when compared to wild-type clones for all axon regions analyzed (Supplementary Fig. 2J). We found that Bsk inactivation also resulted in many changes in the axonal architecture, with the increased presence of large protrusions and swellings (Supplementary Fig. 2B compared to wild type, Fig. 2C–E). Together these results strongly suggest that Bsk loss does not result in an initial defect of axon extension but in the subsequent failure in axon stabilization, leading to neurodegeneration and axon loss.

### Bsk phosphorylation is essential for axonal morphogenesis

**Fig. 3:**
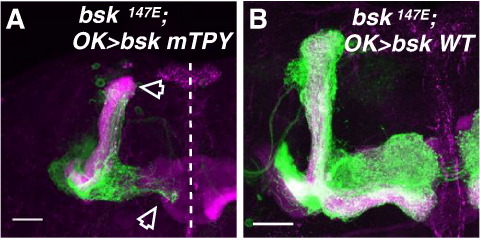
JNK phosphorylation is essential for axonal morphogenesis. (A, B) *bsk^147e^* neuroblast clones in the presence of Bsk mTPY (A), or wild-type Bsk (B). Wild-type Bsk, but not Bsk mTPY, expression rescues the axon phenotype. Cell body sections have been omitted in both panels. Scale bars: 20 μm. Green, CD8-GFP. Magenta, Fas2.

Bsk is activated by phosphorylation by the JNKKs, Hep and MKK4, on two predicted residues on threonine 181 and tyrosine 183 (Glise et al., 1995; Han et al., 1998). We first determined the relevance of these sites by generating a Bsk mutant that removes the phospho-acceptor sites (Bsk T181A, Y183F, or Bsk mTPY). By Western blotting, the phospho-JNK antibody does not detect Bsk mTPY (Supplementary Fig. 3A). Interestingly, overexpression of Bsk mTPY partially mimics the dominant negative (DN) Bsk misexpression phenotype when expressed in wild-type MB neurons (Supplementary Fig. 3B; see below). We determined whether Bsk mTPY expression would rescue the *bsk* phenotype. The results show, in contrast to the wild-type Bsk, Bsk mTPY expression failed to rescue the *bsk* axonal phenotypes, not only in MB neurons but also in optic lobe contralateral projecting (OL) neurons where *bsk* axon extension phenotypes were also observed (Fig. 3 and Supplementary Fig. 3, respectively; quantified in Supplementary Fig. 3G). These results show phosphorylation of these residues are critical for axonal morphogenesis and that the phospho-JNK antibody serves as a valid marker of Bsk activity in these neurons.

### Role of the *Drosophila* JNKK Hep in axonal morphogenesis

**Fig. 4:**
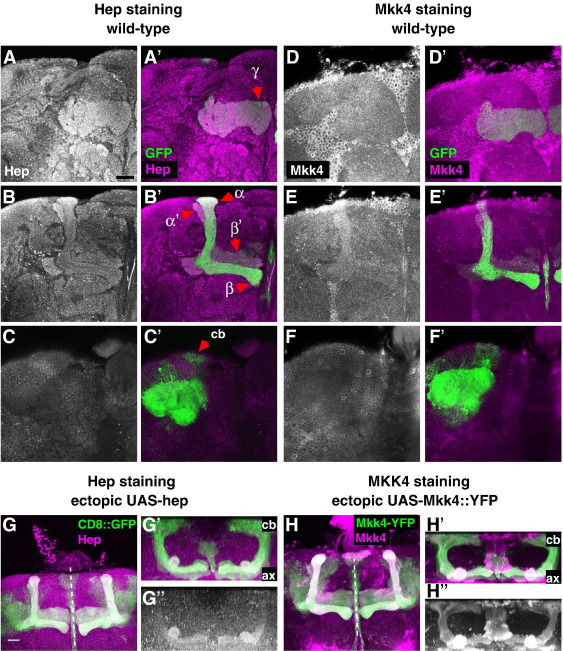
Expression study of JNK kinases Hep and MKK4. (A–F ') Single confocal sections of MB neurons labeled with CD8-GFP and immunostained with Hep (A–C') or Mkk4 (D–F') antibodies. The corresponding panels (A'–F') show overlap between Hep and Mkk4 signals and CD8-GFP labeling. Single sections show γ (A, D), α/β and α'/β' axons (B, E) and MB cell bodies (cb) (C, F), as indicated in A'–C'. (G–G) CD8-GFP labeled MB neurons (green) expressing ectopic Hep (shown in magenta in G and G' and grayscale in G). Dorsal (*y*) projection views (G' and G) show Hep is mainly localized to axons (ax). (H–H) Representative image of MB neurons expressing MKK4YFP (green) and stained with anti-MKK4 (magenta in H and H' and white in H). Dorsal views (H' and H) show MKK4 is localized to axons and cell bodies (cb). Scale bar: 20 μm (*x*-only).

Given the importance of JNK phosphorylation, we next determined the expression pattern of the JNKK, Hep. Hep antibodies were generated and used in western blotting (Supplementary Fig. 4A) and immunohistochemistry (Figs. 4A–C, G). Hep was detected in all MB axons but was less prominent in the cell bodies (Figs. 4A–C). Additional Hep signals were also observed throughout the brain, including the AL. By *UAS-Hep* expression, ectopic Hep staining also showed preferential localization to MB axons (Fig. 4G).

**Fig. 5:**
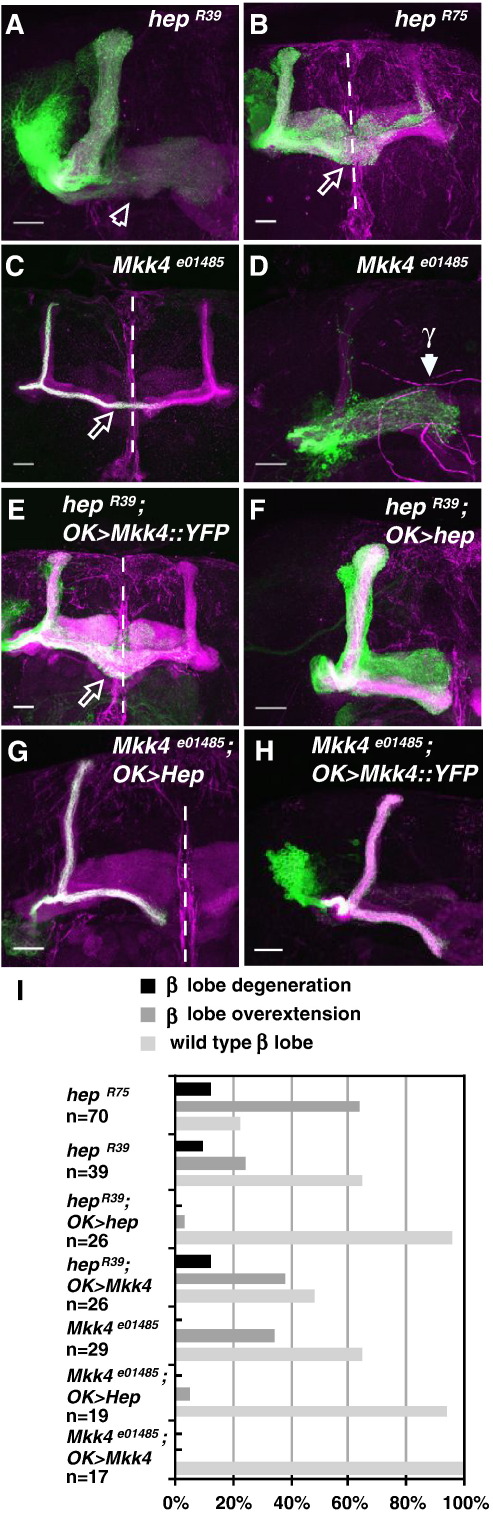
Loss of function of Hep and MKK4 in MB neurons. Representative images of *hep^R39^* (A), *hep^R75^* (B) and *MKK4^e01458^* (C, D) neuroblast clones exhibiting β lobe axon degeneration (A), axon overextension (B, C) and cell proliferation (D) phenotypes. Neuroblast proliferation phenotypes are characterized by the presence of early-born γ neurons and absent later-born α'β' and αβ neurons. (E–H) *hep^R39^*(E, F) and *MKK4^e01458^*(G, H) clones in the presence of ectopic MKK4YFP (E, H) or Hep (F, G). Scale bars: 20 μm. Green, CD8-GFP. Magenta, Fas2. (I) Quantification of the β axon phenotypes. As *Mkk4* leads to proliferation defects in early-born neuroblast clones, later-born αβ neuroblast clones were generated for axon studies. *n*, number of neuroblast clones analyzed.

The role of Hep was determined by loss-of-function analysis. In *hep*-null clones, while axon degeneration was detected, it was not the major phenotype (Figs. 5A, I). Instead, axon overextensions were mainly observed (Figs. 5B, I). This is surprising, given that Hep is consistently described as a central regulator of Bsk (Stronach, 2005), *hep*-null clones might be expected to phenocopy *bsk* phenotypes to the same extent (Fig. 2M).

### Role of *Drosophila* MKK4 in MB neurons

Another JNKK regulator of Bsk is MKK4 (Han et al., 1998). Antibodies to Mkk4 were generated and used in Western blotting and immunohistochemistry (Supplementary Fig. 4B; Figs. 4D–F). Mkk4 was detected in MB axons (particularly in α'β' and αβ axons) and cell bodies (Figs. 4D, E and F, respectively). This was confirmed by the expression of Mkk4-YFP fusion protein in these neurons (Fig. 4H). Using a recently described *MKK4* mutant allele (Thibault et al., 2004) (see Methods), we generated *MKK4*^*e01485*^ MB clones. This also resulted in axon overextension (Fig. 5C), although this phenotype was less frequently observed than in *hep* clones (Fig. 5I). Cell proliferation defects were also observed in neuroblasts derived from earlier-born *MKK4^e01485^*clones (Fig. 5D). This is consistent with recent reports, which show MKK4 loss results in cytokinetic defects in cultured *Drosophila* S2 cells (Bettencourt-Dias et al., 2004; Bjorklund et al., 2006).

Given the similar axon overextension phenotypes, we tested whether Hep and MKK4 are interchangeable. Ectopic Mkk4 was expressed in *hep* clones and *Mkk4* clones were generated with ectopic Hep. *hep* phenotypes were not suppressed by ectopic Mkk4, but the loss of Mkk4 can be rescued by increased Hep (Figs. 5E and G, respectively). In control experiments, these same UAS lines could rescue the corresponding *hep* or *Mkk4* phenotypes (Figs. 5F and H, respectively; quantified in Fig. 5I). These results suggest that while both JNKKs regulate axon growth, Hep plays a more prominent role in these axons (see also Supplementary Fig. 5).

### Partial inactivation of Bsk results in axon overextension

**Fig. 6:**
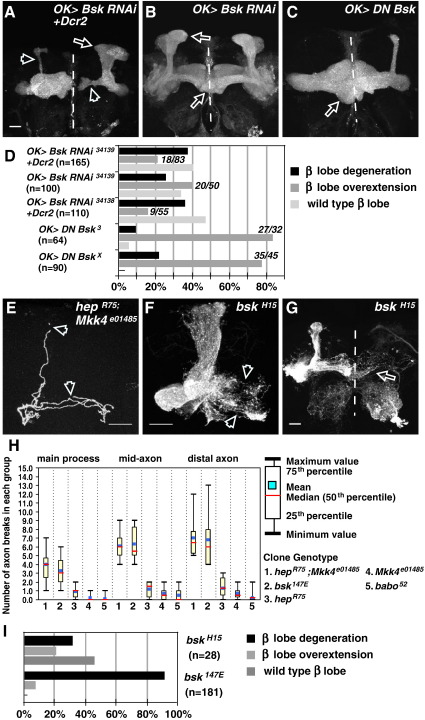
Partial inactivation of Bsk leads to axon overextension. (A, B) MB neurons expressing *Bsk* RNAi. High level of *Bsk* RNAi knockdown leads to a bias in axon degeneration phenotypes (open arrowheads in A), whereas medium RNAi activity levels lead to dorsal axon overgrowth and medial overextensions (open arrows in B). (C) Dominant-negative Bsk (DN Bsk) misexpression resulted in similar phenotypes. We found that pan-MB inactivation of Bsk also resulted in defasciculation defects, characterized by wider, splayed-out axon lobes (for example, the dorsal projection in A, indicated by the open arrow). (D) Quantification of these phenotypes. n, number of hemispheres analyzed. Given that, in many instances, the β-lobe overextension from one hemisphere overlaps against either a similarly overextending, or otherwise wild-type, axon from the contralateral side, we also present a different analysis of the medial projections for all (pan-MB) overexpression genotypes as the number of brains that have β-lobe overextensions over the total number of brains analyzed (in italics next to the relevant bar). Additional analysis and quantifications for dorsal projections are in Supplementary Fig. 6. (E) Representative image of a *hep*^*R75*^, *MKK4*^*e01458*^ mutant single-cell αβ clone. Note axon breaks in the dorsal and medial branch (open arrowheads). Similar breaks were also observed in *bsk^147E^* single-cell αβ clones (data not shown; quantified in H). (F, G) Representative image of *bsk^H15^* neuroblast clones showing axon degeneration (F) and overextension (G) phenotypes. Cell body sections were removed from A, C and F to clearly reveal axon projections. Scale bars: 20 μm. (H) Quantification of axon breaks in *hep*^*R75*^, *MKK4*^*e01458*^ double, *bsk^147E^*, *hep*^*R75*^ or *MKK4*^*e01458*^ or *babo*^*52*^ mutants, as indicated. Ten single-cell αβ clones were analyzed for each genotype. Statistical analysis show a significant difference between *hep*^*R75*^, *MKK4*^*e01458*^ double or *bsk^147E^* clones when compared to *hep*^*R75*^ or *MKK4*^*e01458*^ or *babo*^*52*^ mutants (*P* < 0.05) but no significant difference between *hep*^*R75*^, *MKK4*^*e01458*^ double compared to *bsk^147E^* clones, or between *hep*^*R75*^ and *MKK4*^*e01458*^ or *babo*^*52*^ mutants (*P* > 0.05). The only exception is in the distal axon section of *hep*^*R75*^ axons, where a small proportion of degeneration was observed, as reflected in the *P*-value (0.014). *babo*^*52*^ clones were used as the control in the statistical study. (I) Quantification of the *bsk^H15^* axon phenotypes in neuroblast clones, with a comparison to the null (147E) clones.

As loss of either JNKK results in the same phenotype, one explanation is that partial Bsk inactivity leads to a bias towards axon overextension. To test this, two strategies were used to partially reduce Bsk signals in MB neurons. Given the high level of Bsk in these neurons, we reasoned that either RNAi or DN Bsk misexpression are unlikely to result in a null, but a partial inactivation phenotype. *Bsk* RNAi or DN Bsk expression in all MB neurons resulted in a number of axon defects, with defasciculation, degeneration and overextension phenotypes (Figs. 6A–C). Notably, while *Bsk* RNAi expression resulted in many degenerating axons, axon overextension was also observed, particularly at lower titres of RNAi activity (Figs. 6A, B, respectively; quantified in Fig. 6D and Supplementary Fig. 6A; see Methods). Axon overextensions were also observed by DN Bsk misexpression, and this constituted the major phenotype observed (Figs. 6C, D). Therefore, distinct from the *bsk-null* phenotype where axon degeneration predominates, these results suggest that a partial loss of Bsk leads to axon overextension.

These results, together with the JNKK study, make two further predictions: that concomitant loss of Hep and Mkk4 would result in stronger Bsk inactivity, resulting in degeneration phenotypes, and that hypomorphic *bsk* alleles would lead to less axon degeneration and a higher proportion of axon overextension phenotypes. Indeed, we found that *hep, Mkk* double mutant clones showed a higher frequency of axon breaks than *hep* or *Mkk4* single mutants (Figs. 6E, H). The number of axon breaks in *hep, Mkk* double mutants were comparable to *bsk*-null axons (Fig. 6H; data not shown). We also analyzed the *babo* gene, which when inactivated results in similar axon overextensions (Ng, 2008). We found that *babo* overextended axons had hardly any axon breaks throughout the entire neurite (data not shown; Fig. 6H). This suggests that axon overextension does not necessarily lead to axon breaks and neurodegeneration.

A number of *bsk* hypomorphic alleles were analyzed. *bsk*^*1*^ and *bsk*^*2*^ clones gave wild-type projections (data not shown). *bsk*^*H15*^, which carries a missense mutation in the kinase domain (Berger et al., 2008) resulted in axon degeneration and overextension (Figs. 6F, G, respectively). Our analysis showed while a large fraction of axons were wild type (46.4%), 32.1% gave degeneration phenotypes, while 21.4% exhibited axon overextensions (Fig. 6I). This result shows while the *bsk*-null allele results in predominantly axon degeneration, weaker alleles do result in axon overextension, consistent with the JNKK results.

### Sustained Bsk activity is essential for axon stabilization

**Fig. 7:**
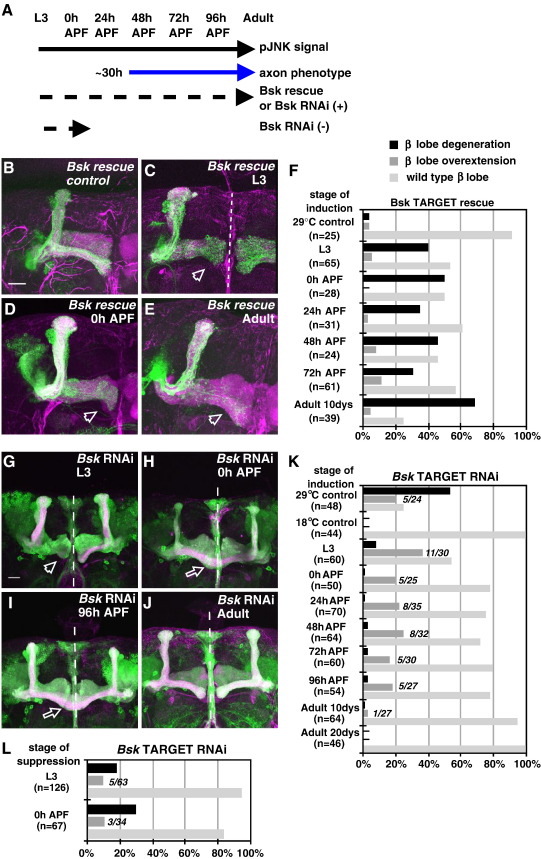
Sustained Bsk levels are essential for axon stability. (A) Bsk activity (as measured by the P-Bsk signal) is detected throughout development and adulthood (black arrow). Yet the axonal phenotypes are observed only at later stages (∼30 h after puparium formation, APF) (blue arrow). To determine the temporal requirements of Bsk-dependent phenotypes, we used the TARGET system to determine whether the induction (+) or suppression (−) of TARGET expression (see schematic in Supplementary Fig. 7A), by transgenic rescue analysis or RNAi at specific stages (larval, pupal or adult) alters the extent of the observed phenotypes. The dashed arrows indicate the extent of the TARGET expression under different temporal and developmental settings. (B–E) Images of *bsk^147e^*neuroblast clones with the Bsk-myc expression under TARGET control. As a control, Bsk TARGET flies raised at 29 °C (GAL4-permissive) throughout exhibited > 90% wild type projections (B). *bsk^147e^*clones with Bsk-myc expression induced at developmental stages L3 (C), and 0h APF (D). (E) *bsk^147e^*clones with Bsk-myc expression restricted only to the adult stage for 10 days post-eclosion. (F) Quantification of these *bsk* rescue phenotypes. Induced at shorter periods, many flies exhibited β-axon degeneration. *n*, number of neuroblast clones analyzed. (G–J) *Bsk* RNAi expressed under TARGET control. *Bsk* RNAi expression induced at stages L3 (G), 0 h (H), or 96 h APF (I) resulted in axon degeneration (G), and axon overextension phenotypes (H, I). (J) *Bsk* RNAi restricted to adult stages for 10–20 days post-eclosion showed wild-type projections. With the exception of adult-stage induced flies, all earlier induced flies were dissected as 3-day adults. Scale bars: 20 μm. Green, CD8-GFP. Magenta, Fas2. (K) Quantification of the *Bsk* TARGET RNAi phenotypes. *n*, number of brain hemispheres analyzed. In K and L, the β-lobe overextension analysis is also presented as the number of overextended brains over the total number of brains analyzed (italics). Note both protocols do not reflect a strict ‘on’ time at the indicated developmental stages but rely on the ‘on’ kinetics of the TARGET system. In our manipulations, we detected expression from 24 h and robustly at 72 h post-induction (Supplementary Fig. 7B–E; data not shown). Therefore, a period of RNAi and Bsk-myc accumulation is required for effective downregulation of *Bsk* mRNA transcripts and suppression of *bsk*-null phenotypes (respectively). (L) Quantification of the *Bsk* RNAi phenotype using a reverse TARGET protocol, with an early induction followed by a suppression of RNAi transgene expression at L3 or 0 h APF, as indicated. Note the increased representation of axon degeneration over overextension phenotypes at the early phase of Bsk inactivity.

Is Bsk activity required throughout MB development and adulthood, as the phospho-JNK antibody results would suggest, or at specific periods? This was investigated by temporally controlling Bsk expression in two ways (Fig. 7A). First, a Bsk rescue experiment was performed. By placing *UAS-Bsk* under the control of the TARGET system (McGuire et al., 2003), raising animals at GAL4-restrictive (18 °C) or GAL4-permissive (29 °C) conditions enabled us to control the timing of Bsk rescue transgene expression in *bsk*-null clones (Fig. 7A and Supplementary Fig. 7; see Methods). The results are summarized in Figs. 7B–E (quantified in F). They show that Bsk activity is required throughout development to completely rescue the axonal phenotypes. Shorter, latent periods of Bsk expression only partially rescued the *bsk-*null phenotypes. In addition, prolonged adult-restricted expression had very little effect.

In a second experiment, a *Bsk RNAi* trangene was expressed also under TARGET control. This enabled us to perform tissue-specific inactivation in a stage-dependent manner. We found that inducing *Bsk* RNAi expression from larval (wandering L3) to late pupal (48–96 h APF) stages resulted in axon overextension and, to a smaller extent, axon degeneration phenotypes (Figs. 7G–J, quantified in K). Prolonged *Bsk* RNAi activity restricted to the adult stage had very little effect, suggesting that Bsk activity is dispensable in adults (Figs. 7J, K). When expressed at shorter, later periods, these axonal phenotypes were also not as frequent as when the *Bsk* RNAi was induced throughout development. This suggests that the ‘full’ Bsk inactivation phenotype reflects an accumulative period of Bsk signaling throughout development (see Discussion).

We also performed a ‘reverse’ protocol with an early induction of *Bsk* RNAi, followed by a suppression of RNAi expression from L3 or 0h APF stages. Similar to the above treatment, this also resulted in axonal phenotypes (Fig. 7L; data not shown). Again, the extent of these phenotypes was not as frequent as when *Bsk* RNAi was induced throughout development. Interestingly, the early treatment resulted in more axon degenerations than overextensions, when compared to the late induction protocol (Fig. 7J), suggesting that the degeneration phenotype is more sensitive to an early phase of Bsk inactivity, while the overextension phenotype is prevented more by a later phase of Bsk.

Together these experiments suggest Bsk activity has to be sustained throughout development to ensure proper axonal morphogenesis.

### A graded AP-1 response regulates Bsk-dependent axon stability

**Fig. 8:**
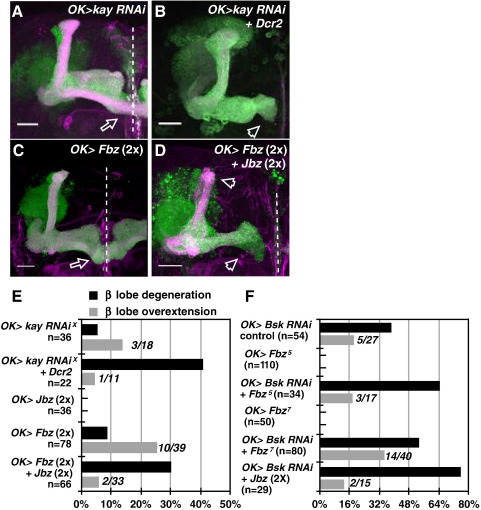
A graded AP-1 signal mediates Bsk responses. (A–D) MB neurons expressing *kay* RNAi (A), *kay* RNAi plus *Dcr2* (B), Fbz (C), or Fbz plus Jbz (D) showed axon overextension (A, C) and degeneration (B, D) phenotypes. Copy numbers of expressed transgenes are indicated in parenthesis. Progeny derived from Fbz and Jbz crosses were raised at 29 °C to increase the possibility of detecting any axonal phenotypes due to misexpression. Scale bars: 20 μm. Green, CD8-GFP. Magenta, Fas2. (E) Quantification of these phenotypes. In E and F, also shown is the β-lobe overextension analysis expressed as the number of overextended brains over the total number of brains analyzed (italics). (F) Genetic interaction assay using *Bsk* RNAi (line 34138) with dominant-negative AP-1. *Bsk* RNAi expressing flies were grown at 29 °C, in presence of one copy of Fbz or two copies of Jbz, showed an enhancement in axonal defects. As controls, *Bsk* RNAi flies were expressed with CD8GFP alone. Single copy expression of Fbz (line 5 or 7) or two copies of Jbz (lines 1 and 10) (E) did not result in gross axon defects. n, number of hemispheres analyzed.

We determined whether Bsk signals are mediated through AP-1, which in *Drosophila* consists of the transcription factors Fos and Jun. Through clonal analysis using strong, null alleles or dominant-negative (Jbz) misexpression, Jun (also known as Jra) inactivation alone had no effect on gross axon morphology (*Jra*^*1*^, *n* = 38 clones, *Jra*^*2*^, *n* = 30 clones, *Jbz*, *n* = 36 hemispheres; both 100% wild type; Supplementary Fig. 8A,B and E, respectively). Loss-of-function analysis on *Fos* (also know as *kayak*, or *kay*) was performed. These axons appeared wild type (*kay*^*1*^, *n* = 21, 100% wild type; *kay*^*ED6315*^, *n* = 30, 93.3% wild type; Supplementary Fig. 7C, D), most likely because true null alleles for *kay* do not exist (Giesen et al., 2003; Weber et al., 2008). Therefore, RNAi and dominant-negative misexpression approaches were used. *Kay* RNAi expression resulted in axon overextension (Fig. 8A). In the presence of Dcr2, stronger *Kay* RNAi resulted in axon degeneration phenotypes (Fig. 8B), and this was the dominant phenotype observed (Fig. 8E). Axon overextensions were also observed when Fbz was misexpressed (Fig. 8C). However, axon degeneration phenotypes were observed when Fbz was co-expressed with Jbz (Fig. 8D; quantified in E). To test whether these AP-1 phenotypes are linked to Bsk, we performed genetic interaction assays by expressing *Bsk* RNAi together with Jbz or Fbz (data not shown; Fig. 8F). We found that the *Bsk* RNAi effect was strongly enhanced by single copy expression of Fbz, and by two copies of Jbz.

These results suggest the AP-1 complex mediates the Bsk response in MB neurons. These signals were similarly graded, whereby weak AP-1 inactivation leads to axon overextension but stronger loss results in a bias towards axon degeneration, with Kay playing the major role in these neurons.

## Discussion

This study shows that the JNK pathway regulates distinct aspects of axonal morphogenesis, namely to prevent axon overextension and axonal degeneration. To understand the underlying differences, we showed that the level of Bsk activity, its duration and the developmental phase of an organism determine how developing axons respond to these signals. The JNK signals are converted into a transcriptional response through AP-1 and that these signals are similarly graded in axonal morphogenesis.

The MAPK family of proteins are involved in many aspects of cellular behavior and several hypotheses have been put forward to account for their diverse action. Previous in vitro studies show signal duration and signal strength can bias MAPK responses. This has been studied mainly in the context of Erk responses towards cell proliferation and differentiation in cultured cells (Marshall, 1995; Murphy and Blenis, 2006). How these parameters regulate Erk signals have been explored using experimental and theoretical approaches (Kholodenko, 2006; Murphy and Blenis, 2006). Below we discuss the results of this study and focus on the parameters that regulate JNK-dependent axonal stability in vivo.

Our study explains how a single protein kinase can potentially regulate multiple aspects of neuronal morphogenesis. Signals that regulate Bsk activity can generate distinct axonal phenotypes depending on the signaling network, strength and duration. The JNKK signal network is linked to signal strength, since both JNKKs converge on Bsk and determine its relative activity levels (Supplementary Fig. 9A). In the case where the signal ensures axons do not overextend, both Hep and Mkk4 are essential as the loss of either input leads to axon overextension. This is phenocopied by hypomorphic conditions of Bsk. However, for signals that protect against neurodegeneration and axonal loss, the JNKKs seem to act redundantly, as loss of either JNKK does not result in axon degeneration, whereas loss of Bsk, or Hep together with Mkk4 does. The bias in responses suggest while lower levels of Bsk activity are sufficient to protect against axon degeneration, the mechanism that protects against axon overextension requires higher threshold levels of Bsk activity. In both cases, Bsk phosphorylation is critical for as loss of their sites renders Bsk fully inactive.

What is currently unknown is the nature of the signal(s) that regulate the JNK responses in these neurons, although it is likely to be active throughout development. Also unclear is whether the signal that protects against neurodegeneration is linked to axon overextension, although we show overextended axons *per se* do not necessarily lead to axon breaks. In one scenario, these could be represented by distinct molecules and signaling mechanisms: one signal acts to prevent degeneration, while another stabilizes axons at the correct termini by preventing overextension. In a distinct scenario, this may be one and the same factor, which acts to promote both axon stability and termination at the correct target site. Nonetheless, our results suggest these responses require different Bsk threshold activities.

The phospho-JNK staining results show Bsk is active throughout MB development and adulthood (Supplementary Fig. 9B). The genetic results show, for axonal morphogenesis, sustained Bsk activity is essential only throughout development, but it is not restricted to a narrow, critical period within development. As Bsk activity (or inactivity) during adulthood does not change the pre-existing axonal morphology of these genotypes, this suggests JNK-dependent physiology changes between development and adulthood, and the adult-stage phospho-JNK signals most likely reflect a distinct mode of MB regulation. This switch may be an example of a cell-context requirement of JNK signaling at different developmental stages. Although yet to be defined, one possibility is an adult role in synaptic plasticity and growth, as shown in *Drosophila* NMJ studies (Collins et al., 2006; Sanyal et al., 2002).

We found that shorter periods of Bsk inactivity and activity tend to result in a weaker effect (as opposed to no effect at all) compared to protocols where *Bsk* RNAi or rescue activity is ‘on’ throughout development. Based on these results, we propose, for maintaining optimal axon stability, Bsk signals are read ‘additively’ throughout development (Supplementary Fig. 9B). Rather than as a ‘temporal summation’ module, where signals need to reach a timed threshold level of activity to evoke ‘all-or-none’ response, we find the shorter periods of Bsk activity can still derive a (albeit sub-optimal) morphogenetic response. Nonetheless, there is still an activity threshold required to protect against degeneration (relatively low) and axon overextension (relatively high).

Note that in our genetic paradigm JNK activity protects against neurodegeneration and axonal loss. In many studies, JNK activity provokes these effects, in response to physical injury, genetic, environmental and stress stimuli (Miller et al., 2009; Morfini et al., 2006; Waetzig et al., 2006). One previous study in mutant mice showed that JNK inactivation also leads to axonal loss. These studies also showed JNK loss does not affect initial axon commissure formation (Chang et al., 2003). Interestingly, JNK and AP-1 activity is often upregulated in response to nerve injury and thought to be essential for axonal repair post-injury (Herdegen et al., 1998; Raivich et al., 2004). How our study relates to these models of disease, injury and repair remains speculative, however, it shows parameters such as activity levels, timing and developmental stages may be key to understanding JNK-dependent dysfunctions in the nervous system.

Our study also raises the possibility that JNK signals are related to developmentally regulated degenerative events such as axonal pruning (Lee et al., 1999). However, this is unlikely, as our results show (1) all adult *bsk* γ-axons exhibit only a single, medial projection (Fig. 2J), showing that mutant axons prune properly, (2) neither is there a delay in pruning (Figs. 2G–I), as aberrant, non-pruned, dorsal projections are not visible at mid-pupal stages, when pruning (followed by re-extension) has completed (Lee et al., 1999), and (3) forced ectopic activation of the JNK pathway also does not block axon pruning (Supplementary Fig. 10). Together these results suggest Bsk signals (its loss or gain) does not affect developmental pruning in MB neurons.

Our results show AP-1 signals are similarly graded to these distinct phenotypes (Supplementary Fig. 9A). How predominantly axon-based Bsk signals are translated into a graded, nuclear-based AP-1 transcriptional readout awaits further investigation. One can potentially envisage that the different axonal behaviors derived from graded AP-1 responses result from gene expression programs involving separate pools of immediate early gene products, or the same set of transcriptional targets expressed in different quantities, and/or perhaps even under different temporal settings.

Currently, it is unclear how the Bsk/AP-1 signaling program regulates these changes in axonal behaviors. Previous studies show Bsk signals result in transcriptional responses involving distinct gene targets linked to the actin cytoskeleton, cell adhesion, oxidative stress, extracellular matrix, autophagy, cell cycle and apoptotic control (Homsy et al., 2006; Hyun et al., 2006; Jasper et al., 2001; Moreno et al., 2002; Uhlirova and Bohmann, 2006; Wang et al., 2003; Wu et al., 2009). Many of these processes control Bsk-regulated morphogenesis. In our preliminary analysis, we found Bsk/AP-1 signals do lead to changes in the actin and microtubule cytoskeleton, axonal transport and caspase-related activities (unpublished observations). These preliminary results may be related to the axonal breaks and to changes in the axonal architecture (such as swellings, filopodia and lamellipodia protrusions) that were observed in *bsk* single-cell clones.

While we show AP-1 mechanisms are involved, it is likely that not all of these cellular changes are AP-1 related. For example, the axonal swellings observed in *bsk* clones may arise from defects in axonal transport, which was recently reported to be perturbed in different JNK settings (Horiuchi et al., 2007), and our preliminary results show many of these swellings do contain an accumulation of axonal transport cargo, such as organelles and vesicles, as measured using mitochondrial and synaptic markers. Many AP-1-independent targets have been reported in mammalian JNK studies and direct JNK regulation of the actin and microtubule cytoskeleton has been proposed (Bjorkblom et al., 2005; Chang et al., 2003; Gdalyahu et al., 2004; Huang et al., 2003; Tararuk et al., 2006; Yoshida et al., 2004). Currently, it is unclear how relevant these targets are in Bsk responses in vivo. In some instances, these targets are not relevant as either there are no obvious homologs in Drosophila (such as DCX), or the fly proteins do not have the equivalent JNK phospho-regulatory sites (such as paxillin).

In summary, our results highlight the signaling mechanisms that control the JNK pathway during neuronal morphogenesis. The basis of distinct morphogenetic functions can be accounted by the graded levels by which JNK input signals are mediated and the way AP-1 transcriptional output signals are generated, as well as the duration of the signal propagation within developing neurons.

## Appendix A

Supplementary Figure 1 . Bsk inactivation resulted in a downregulation of the phospho-Bsk and Bsk signal in MB neurons. **(A-A’’)** In wholemount brains, wild-type MB neurons, labeled with the CD8-GFP (A), and immunostained with anti-phospho JNK (A’) and anti-JNK1 (A’’) showed high levels of P-JNK/JNK signals. **(B-C’’)** In identical immunostaining protocols, MB neurons expressing *Bsk* RNAi (B) or DN Bsk (C) showed a strong reduction in P-JNK levels (B’,C’). Loss of JNK signals was observed by *Bsk* RNAi treatment (B’’), together with the expected increase in JNK signals due to ectopic DN Bsk expression (C’’). High P-JNK activity was also observed in other brain regions such as the antennal lobe (AL) and ellipsoid body (eb), as indicated. Scale bar: 50 μm.

Supplementary Figure 2 . Bsk inactivation result in neurodegeneration and the increased presence of axonal swellings and protrusions. **(A)** A *bsk*^*147e*^ neuroblast clone exhibiting an infrequently observed axon overextension phenotype (open white arrow). **(B)** Image of two *bsk^147e^*single cell clones. Magnified images of the proximal **(B’)** and distal MB axon regions **(B’’)** indicate the presence of large protrusions (blue arrows) and swellings (blue arrowheads). The open white arrow in B’’ shows a single axon overextended from the contralateral hemisphere (right). **(C-E)** Image of wild-type (*FRT*^*40A*^) Kenyon cells (labeled by the asterisks) (C) and representative images of the middle (D) and distal axon regions (E). Wild-type MB single cell clones do not reveal the presence of swelling, protrusions or breaks. **(F-G)***bsk*^*147e*^ single cell clones also show breaks (yellow arrowheads in **F**) and thinning (yellow arrow in **G**) in the main process close to the cell body, which were rarely observed in wild-type neurons **(H**, quantified in **J)**. Dashed line, midline. Scale bars: 20 μm (A–E), 10 μm (F–H). Green, CD8-GFP. Magenta, Fas2. **(I)** Quantification of all MB axon projections defects observed in MB *bsk*^*147e*^ adult neuroblast clones. **(J)** A box plot showing the neurodegeneration phenotypes (breaks and thinning) in *bsk*^*147E*^ mutant and wild-type γ single cell clones. Three regions were analyzed: main process (cell body to primary branch), mid- and distal axonal process. In each case, the probability (*P*) values (paired *t*-test) show a significant difference between wild-type and *bsk* neurons. Each box plot gives the mean (blue square), the median (red line), 25^th^ percentile and 75^th^ percentile, maximum and minimum values. The sample size of each group is 10 neurons.

Supplementary Figure 3 . Bsk expression analysis in vitro and in vivo shows the critical role of JNKK phosphorylation sites. **(A)***Drosophila* S2 cells were transfected with pUAST *Bsk-*myc or pUAST *Bsk*-myc^*T181A, Y183F*^ (Bsk-myc mTPY), as indicated (**+**)*.* Transfected cells lysates were subjected to SDS-PAGE and Western blot analysis with anti-phospho JNK and JNK antibodies, as shown. Molecular weight markers shown are in kDa. **(B-B’’)** Representative image of CD8-GFP labeled MB neurons (green) expressing the phospho-inactive Bsk-myc mTPY, immunostained with anti-myc (magenta in B’ and B’’). Note the presence of axon overextension (white open arrow), which was also observed by DN Bsk misexpression (see Results). **(C-F)** CD8-GFP labeled wild-type (C), *bsk^147e^*(D), or *bsk^147e^*optic lobe (OL) neuroblast clones with ectopic Bsk WT (E) or Bsk mTPY (F), as indicated. Dashed blue arrows indicate defective OL axon termination points in *bsk*^*147e*^ genotypes in the central brain region. Scale bars: 20 μm. **(G)** Quantification of the MB and OL *bsk*^*147e*^ axonal phenotypes. n, number of neuroblast clones analyzed.

Supplementary Figure 4 . JNKK expression analysis of Hep and Mkk4 using anti-Hep and Mkk4 antibodies. **(A-B)** S2 cells were transfected with pUAST Hep-RFP (A) or pUAST MKK4YFP (B). Transfected cell lysates were subjected to SDS-PAGE and immunoblot analysis, using anti-Hep (A) and anti-Mkk4 antibodies (B). Molecular weight markers are in kDa. The asterisk indicates a degradation product derived from Hep-RFP.

Supplementary Figure 5 . Analysis of p-JNK levels upon the loss of JNKK expression by RNAi. We tried to determine the contribution of Hep and Mkk4 activities on p-Bsk levels in MB neurons *in situ*. **(A-B’)** Expression of *Hep* RNAi (with Dicer2) resulted in a strong axon β-lobe overextension phenotype (open arrow), together with the frequent loss of the dorsal lobe (A,B), consistent with the *hep* clonal phenotype (Fig. 5B; data not shown). By wholemount antibody staining, a strong reduction in p-JNK levels in MB neurons was also observed (A’,B’). A reduction in Hep protein levels was also observed in *Hep* RNAi, however, a residual amount of Hep protein may still be present, as determined by Hep antibody staining (data not shown). **(C-D’)** Expression of *Mkk4* RNAi (with Dicer2) resulted in a weaker axon β-lobe overextension phenotype (open arrow in C,D), consistent with the *Mkk4* clonal phenotype (Fig. 5C). This was not consistently observed. We also did not see a strong reduction in p-JNK levels (C’,D’). Even though *Hep* RNAi brains were imaged at a higher detector gain and power setting, reduced p-JNK levels in MB neurons were more visible (A’,B’), compared to *Mkk4* RNAi brains (C’,D’). While we can conclude that Hep contributes substantially to p-Bsk levels, given that RNAi efficacy can vary (due to positional effects of the transgene insertion site, dsRNA design and possible differences in JNKK protein stabilities), the contribution of Mkk4 on p-Bsk level has to be investigated further to determine whether Mkk4 truly plays a less prominent role in Bsk signaling. Panels A, A’, C and C’ are confocal images from a single section. Panels B, B’, D and D’ show the entire *z*-projection stack of these same MB neurons. Scale bar, 20 μm.

Supplementary Figure 6 . Analysis of MB axon projection defects in different Bsk loss-of-function settings. **(A)** Additional analysis on the effect of ectopic Dcr2 on the *Bsk* RNAi phenotype. The experiment was performed five times, and the *P-*values indicated are for the α-lobe degeneration, and β-lobe degeneration and β−overextension phenotype. The presence of ectopic Dcr2 is indicated (**+**). **(B**) Quantification of dorsal α-projection defects in *Bsk* RNAi and DN Bsk expressing MB neurons.

Supplementary Figure 7 . Analysis of TARGET induction in MB neurons. **(A)** A schematic of the TARGET experiments using a Bsk rescue transgene (expressed in *bsk* clones), or Bsk RNAi expression. In this system, the conventional GAL4-UAS system is conditionally regulated by a temperature sensitive allele of GAL80 (GAL80ts). At 18 °C, GAL4 transcriptional activity is repressed by GAL80ts, thus preventing the expression of the desired transgene, whereas this repression is relieved by a temperature shift to 29 °C, since GAL80ts becomes inactivated. This allows us to control GAL4 activity in MB neurons in a temporal, stage-dependent manner. Adapted from (McGuire et al., 2003). **(B-E)** MB CD8-GFP expression controlled under the TARGET system. (B) No CD8-GFP expression was detected in flies cultured at 18 °C throughout (Gal4-restrictive). At GAL4-permissive, 29 °C, CD8-GFP expression is increasingly detected at 24 hr (C), 48 hrs (D) and 72 hrs (E) post-induction. Scale bar: 20 μm. Green, CD8-GFP epifluorescence. Magenta, Fas2 staining.

Supplementary Figure 8 . Inactivation of Jun and Fos in MB neurons. **(A-D)** Representative images of *Jra*^*1*^ (A), *Jra*^*2*^ (B) *kay^1^*(D) and *kay^ED6315^*(D) MB neuroblast clones show wild type projections. As *kay*^*1*^ early-born neuroblast clones have cell proliferation defects, later-born αβ neuroblast were used in the analysis. **(E)** MB neurons misexpressing two copies of dominant-negative *Jun* (*Jbz*). Cell body sections were removed from E to clearly reveal axon projections. Scale bars: 20 μm. Green, CD8-GFP. Magenta, Fas2.

Supplementary Figure 9 . The role activity level and signal duration plays in Bsk signaling in neurons. A working model of Bsk-dependent axon stability derived from this study. **(A)** Hep and Mkk4 activities determine the level of Bsk/AP-1 signals in MB neurons resulting in wild-type (normal activity), axon overextension (partial activity), or degenerating (completely loss of Bsk/AP-1) axons. **(B)** A temporal model of Bsk-dependent axonal morphogenesis. Bsk activity (measured by the P-Bsk signal) is detected throughout development and adulthood (up to 14 days post-eclosion; unpublished observations). Our genetic results show, to maintain optimal axonal stability, Bsk is required throughout the pre-adult, developmental phase. Furthermore, the quantification studies from Bsk TARGET RNAi and rescue analysis show these signals act additively (see Discussion).

Supplementary Figure 10 . Forced expression of constitutively active JNK signals does not block MB axon pruning. **(A)** Ectopic expression of activated JNKK (HepCA) in γ-neurons (analyzed in single-cell clones) does not result in a block in developmental pruning. **(B)** For a comparison, loss of Smad signals (*Smad2*, or *Smox*-Flybase) results in axon pruning defects, characterized by the presence of larval-stage dorsal and medial γ projections at the adult stage (Zheng et al., 2003). Normal adult γ projections typically have a single medial projection, as seen in *bsk-*null (Fig. 2J) and HepCA-expressing clones. Scale bar: 20 μm. In both images the cell body sections have been remove to clearly reveal the axonal projections.
