# Supplementary figures and images for: Signal strength and signal duration define two distinct aspects of JNK-regulated axon stability
Source: Dev Biol. 2010 Mar 1;339(1-3):65–77. doi: 10.1016/j.ydbio.2009.12.016 (PMC2845820; doi:10.1016/j.ydbio.2009.12.016)

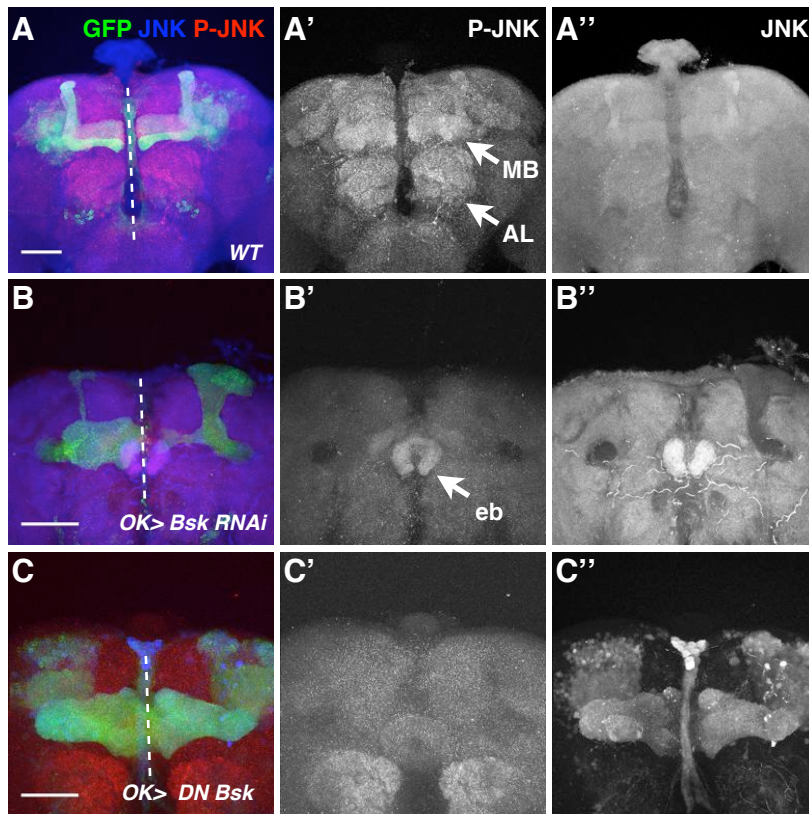

**Supplementary  
Figure 1  
Rallis**

Supplement: Supplementary Figure 1 — . Bsk inactivation resulted in a downregulation of the phospho-Bsk and Bsk signal in MB neurons. (A-A’’) In wholemount brains, wild-type MB neurons, labeled with the CD8-GFP (A), and immunostained with anti-phospho JNK (A’) and anti-JNK1 (A’’) showed high levels of P-JNK/JNK signals. (B-C’’) In identical immunostaining protocols, MB neurons expressing Bsk RNAi (B) or DN Bsk (C) showed a strong reduction in P-JNK levels (B’,C’). Loss of JNK signals was observed by Bsk RNAi treatment (B’’), together with the expected increase in JNK signals due to ectopic DN Bsk expression (C’’). High P-JNK activity was also observed in other brain regions such as the antennal lobe (AL) and ellipsoid body (eb), as indicated. Scale bar: 50 μm. [file mmc1.pdf]

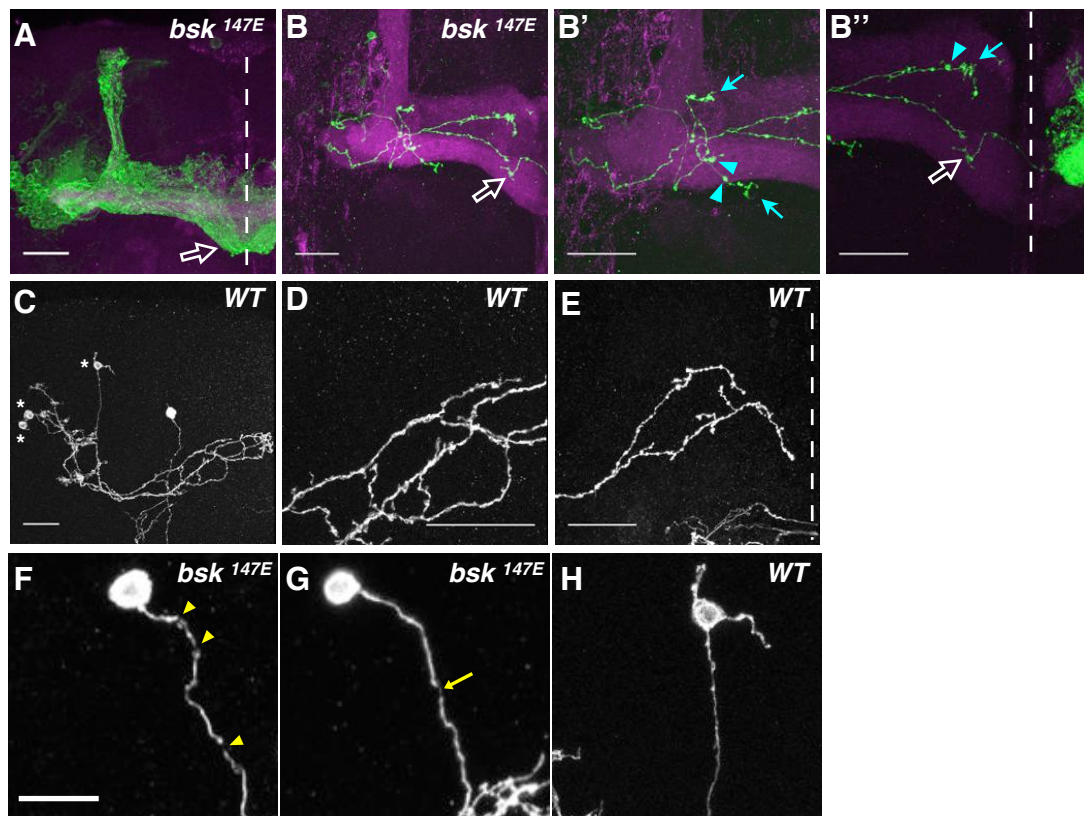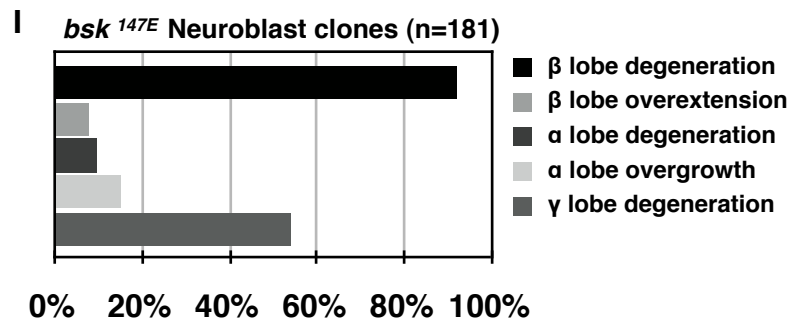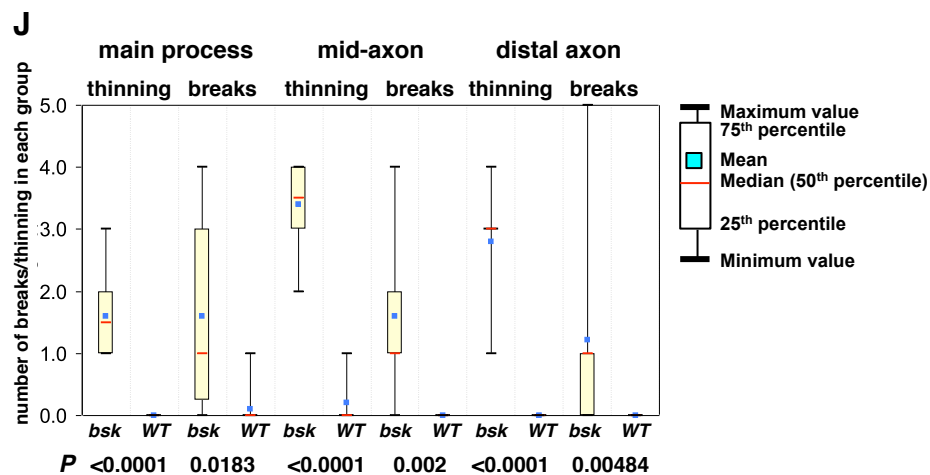

Supplementary  
Figure 2  
Rallis

Supplement: Supplementary Figure 2 — . Bsk inactivation result in neurodegeneration and the increased presence of axonal swellings and protrusions. (A) A bsk147e neuroblast clone exhibiting an infrequently observed axon overextension phenotype (open white arrow). (B) Image of two bsk147esingle cell clones. Magnified images of the proximal (B’) and distal MB axon regions (B’’) indicate the presence of large protrusions (blue arrows) and swellings (blue arrowheads). The open white arrow in B’’ shows a single axon overextended from the contralateral hemisphere (right). (C-E) Image of wild-type (FRT40A) Kenyon cells (labeled by the asterisks) (C) and representative images of the middle (D) and distal axon regions (E). Wild-type MB single cell clones do not reveal the presence of swelling, protrusions or breaks. (F-G)bsk147e single cell clones also show breaks (yellow arrowheads in F) and thinning (yellow arrow in G) in the main process close to the cell body, which were rarely observed in wild-type neurons (H, quantified in J). Dashed line, midline. Scale bars: 20 μm (A–E), 10 μm (F–H). Green, CD8-GFP. Magenta, Fas2. (I) Quantification of all MB axon projections defects observed in MB bsk147e adult neuroblast clones. (J) A box plot showing the neurodegeneration phenotypes (breaks and thinning) in bsk147E mutant and wild-type γ single cell clones. Three regions were analyzed: main process (cell body to primary branch), mid- and distal axonal process. In each case, the probability (P) values (paired t-test) show a significant difference between wild-type and bsk neurons. Each box plot gives the mean (blue square), the median (red line), 25th percentile and 75th percentile, maximum and minimum values. The sample size of each group is 10 neurons. [file mmc2.pdf]

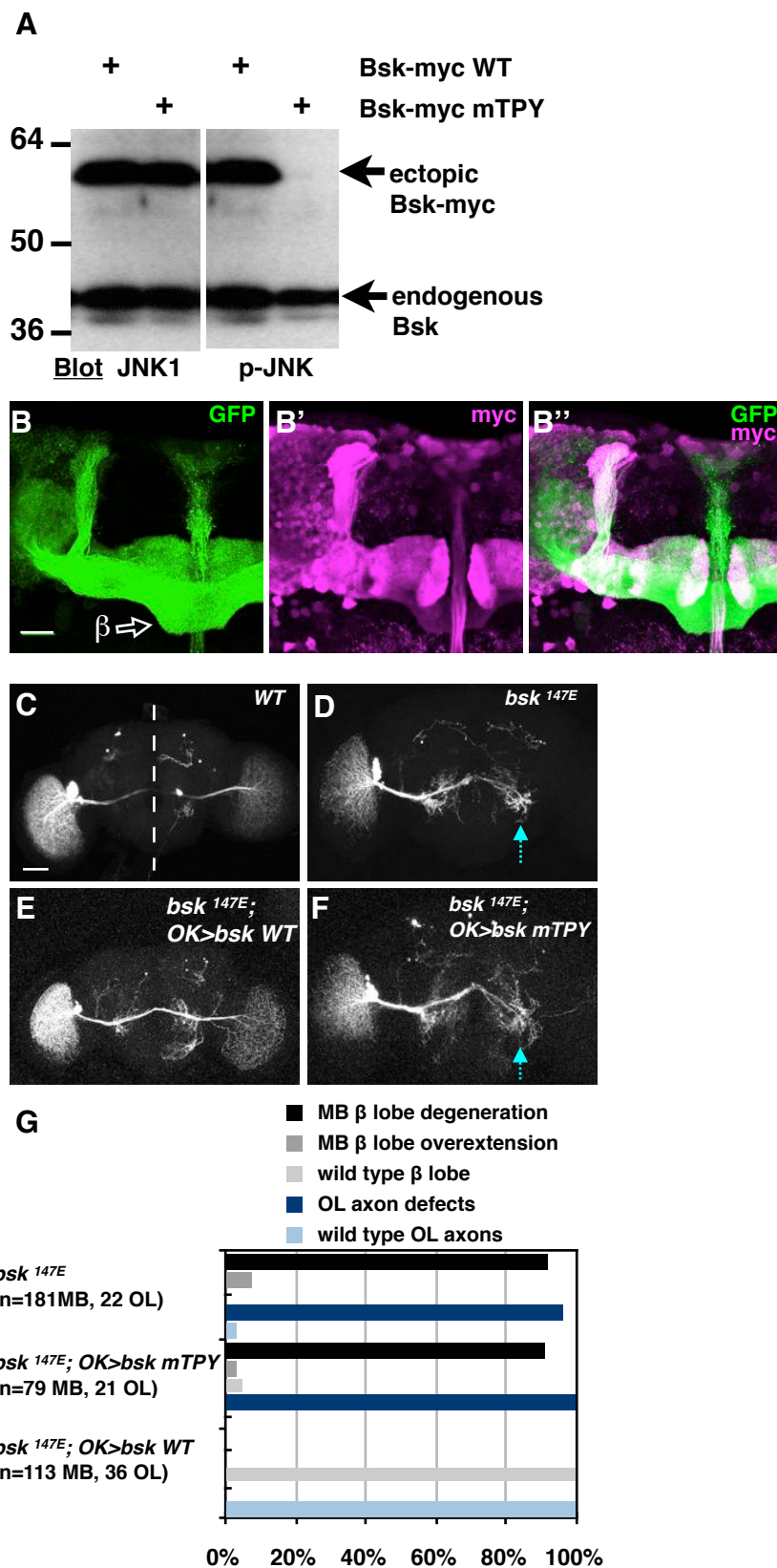

**Supplementary  
Figure 3  
Rallis**

Supplement: Supplementary Figure 3 — . Bsk expression analysis in vitro and in vivo shows the critical role of JNKK phosphorylation sites. (A)Drosophila S2 cells were transfected with pUAST Bsk-myc or pUAST Bsk-mycT181A, Y183F (Bsk-myc mTPY), as indicated (+). Transfected cells lysates were subjected to SDS-PAGE and Western blot analysis with anti-phospho JNK and JNK antibodies, as shown. Molecular weight markers shown are in kDa. (B-B’’) Representative image of CD8-GFP labeled MB neurons (green) expressing the phospho-inactive Bsk-myc mTPY, immunostained with anti-myc (magenta in B’ and B’’). Note the presence of axon overextension (white open arrow), which was also observed by DN Bsk misexpression (see Results). (C-F) CD8-GFP labeled wild-type (C), bsk147e(D), or bsk147eoptic lobe (OL) neuroblast clones with ectopic Bsk WT (E) or Bsk mTPY (F), as indicated. Dashed blue arrows indicate defective OL axon termination points in bsk147e genotypes in the central brain region. Scale bars: 20 μm. (G) Quantification of the MB and OL bsk147e axonal phenotypes. n, number of neuroblast clones analyzed. [file mmc3.pdf]

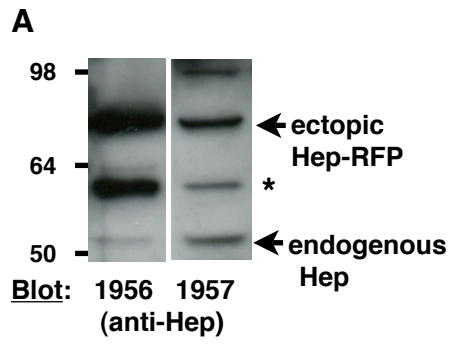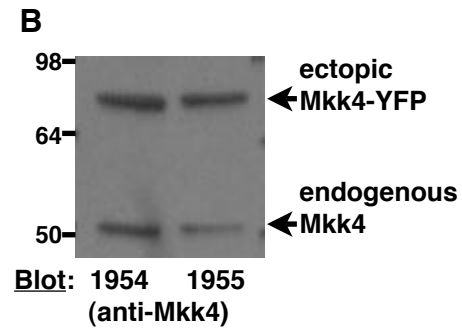

**Supplementary  
Figure 4  
Rallis**

Supplement: Supplementary Figure 4 — . JNKK expression analysis of Hep and Mkk4 using anti-Hep and Mkk4 antibodies. (A-B) S2 cells were transfected with pUAST Hep-RFP (A) or pUAST MKK4YFP (B). Transfected cell lysates were subjected to SDS-PAGE and immunoblot analysis, using anti-Hep (A) and anti-Mkk4 antibodies (B). Molecular weight markers are in kDa. The asterisk indicates a degradation product derived from Hep-RFP. [file mmc4.pdf]

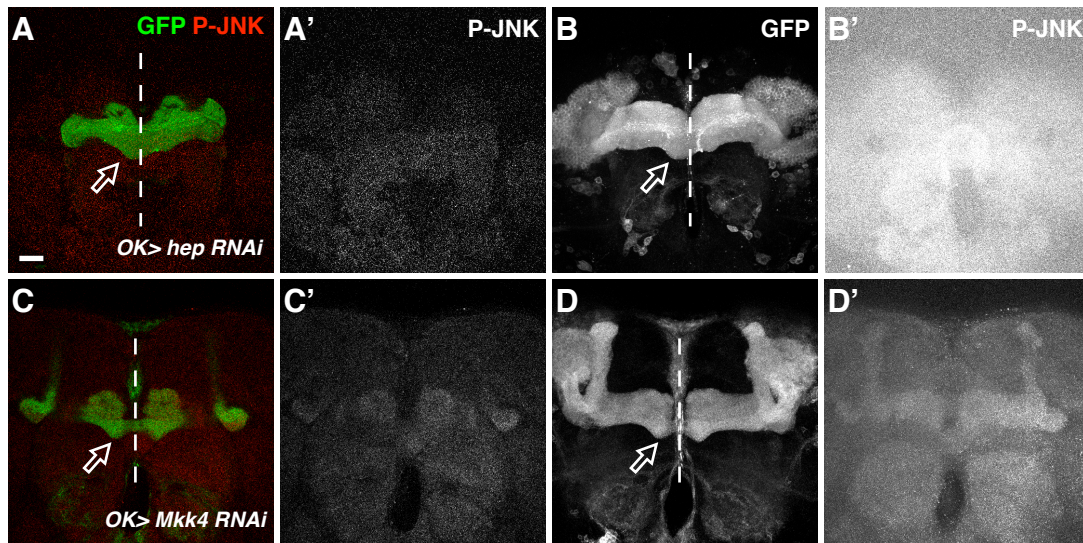

**Supplementary  
Figure 5  
Rallis**

Supplement: Supplementary Figure 5 — . Analysis of p-JNK levels upon the loss of JNKK expression by RNAi. We tried to determine the contribution of Hep and Mkk4 activities on p-Bsk levels in MB neurons in situ. (A-B’) Expression of Hep RNAi (with Dicer2) resulted in a strong axon β-lobe overextension phenotype (open arrow), together with the frequent loss of the dorsal lobe (A,B), consistent with the hep clonal phenotype (Fig. 5B; data not shown). By wholemount antibody staining, a strong reduction in p-JNK levels in MB neurons was also observed (A’,B’). A reduction in Hep protein levels was also observed in Hep RNAi, however, a residual amount of Hep protein may still be present, as determined by Hep antibody staining (data not shown). (C-D’) Expression of Mkk4 RNAi (with Dicer2) resulted in a weaker axon β-lobe overextension phenotype (open arrow in C,D), consistent with the Mkk4 clonal phenotype (Fig. 5C). This was not consistently observed. We also did not see a strong reduction in p-JNK levels (C’,D’). Even though Hep RNAi brains were imaged at a higher detector gain and power setting, reduced p-JNK levels in MB neurons were more visible (A’,B’), compared to Mkk4 RNAi brains (C’,D’). While we can conclude that Hep contributes substantially to p-Bsk levels, given that RNAi efficacy can vary (due to positional effects of the transgene insertion site, dsRNA design and possible differences in JNKK protein stabilities), the contribution of Mkk4 on p-Bsk level has to be investigated further to determine whether Mkk4 truly plays a less prominent role in Bsk signaling. Panels A, A’, C and C’ are confocal images from a single section. Panels B, B’, D and D’ show the entire z-projection stack of these same MB neurons. Scale bar, 20 μm. [file mmc5.pdf]

**A**

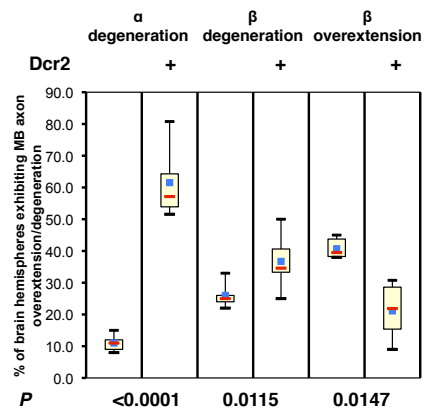

**B**

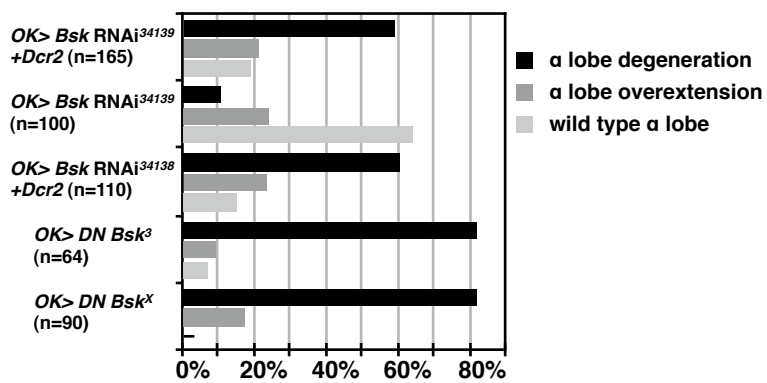

**Supplementary  
Figure 6  
Rallis**

Supplement: Supplementary Figure 6 — . Analysis of MB axon projection defects in different Bsk loss-of-function settings. (A) Additional analysis on the effect of ectopic Dcr2 on the Bsk RNAi phenotype. The experiment was performed five times, and the P-values indicated are for the α-lobe degeneration, and β-lobe degeneration and β−overextension phenotype. The presence of ectopic Dcr2 is indicated (+). (B) Quantification of dorsal α-projection defects in Bsk RNAi and DN Bsk expressing MB neurons. [file mmc6.pdf]

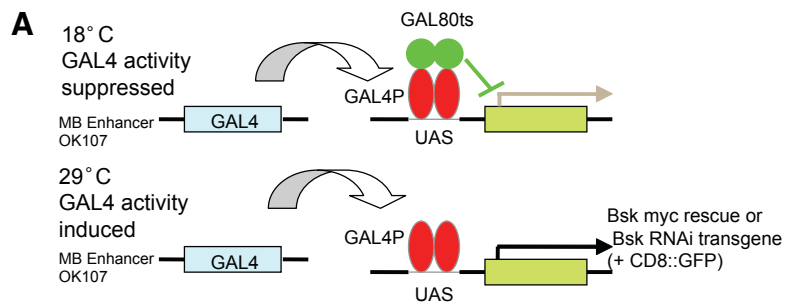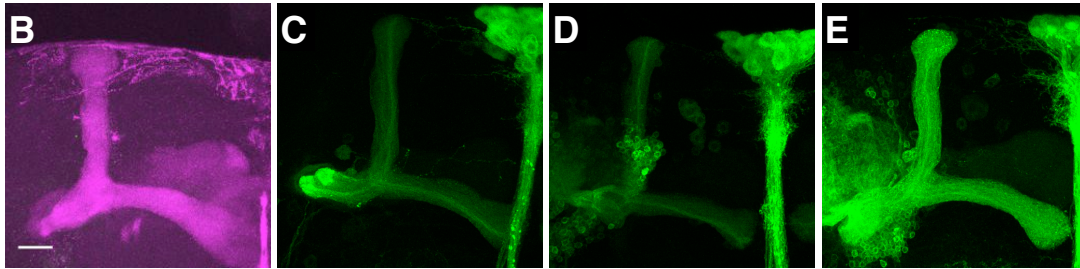

**Supplementary  
Figure 7  
Rallis**

Supplement: Supplementary Figure 7 — . Analysis of TARGET induction in MB neurons. (A) A schematic of the TARGET experiments using a Bsk rescue transgene (expressed in bsk clones), or Bsk RNAi expression. In this system, the conventional GAL4-UAS system is conditionally regulated by a temperature sensitive allele of GAL80 (GAL80ts). At 18 °C, GAL4 transcriptional activity is repressed by GAL80ts, thus preventing the expression of the desired transgene, whereas this repression is relieved by a temperature shift to 29 °C, since GAL80ts becomes inactivated. This allows us to control GAL4 activity in MB neurons in a temporal, stage-dependent manner. Adapted from (McGuire et al., 2003). (B-E) MB CD8-GFP expression controlled under the TARGET system. (B) No CD8-GFP expression was detected in flies cultured at 18 °C throughout (Gal4-restrictive). At GAL4-permissive, 29 °C, CD8-GFP expression is increasingly detected at 24 hr (C), 48 hrs (D) and 72 hrs (E) post-induction. Scale bar: 20 μm. Green, CD8-GFP epifluorescence. Magenta, Fas2 staining. [file mmc7.pdf]

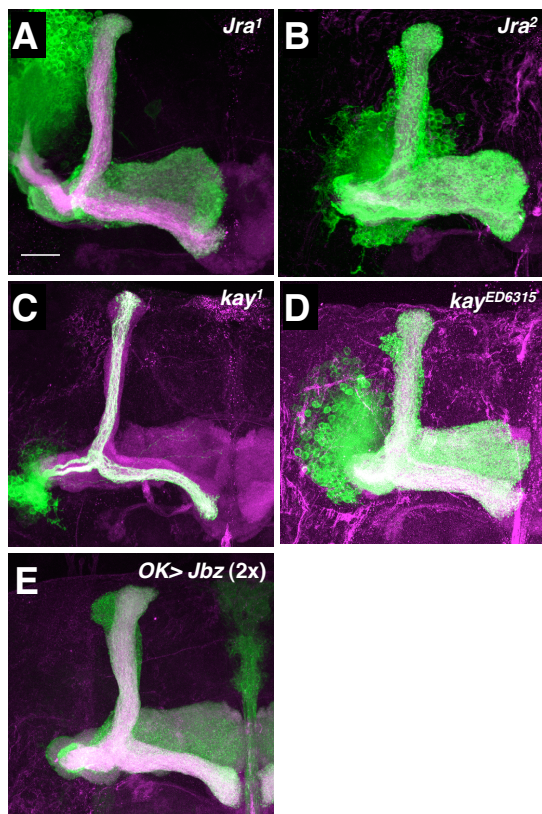

**Supplementary  
Figure 8  
Rallis**

Supplement: Supplementary Figure 8 — . Inactivation of Jun and Fos in MB neurons. (A-D) Representative images of Jra1 (A), Jra2 (B) kay1(D) and kayED6315(D) MB neuroblast clones show wild type projections. As kay1 early-born neuroblast clones have cell proliferation defects, later-born αβ neuroblast were used in the analysis. (E) MB neurons misexpressing two copies of dominant-negative Jun (Jbz). Cell body sections were removed from E to clearly reveal axon projections. Scale bars: 20 μm. Green, CD8-GFP. Magenta, Fas2. [file mmc8.pdf]

A

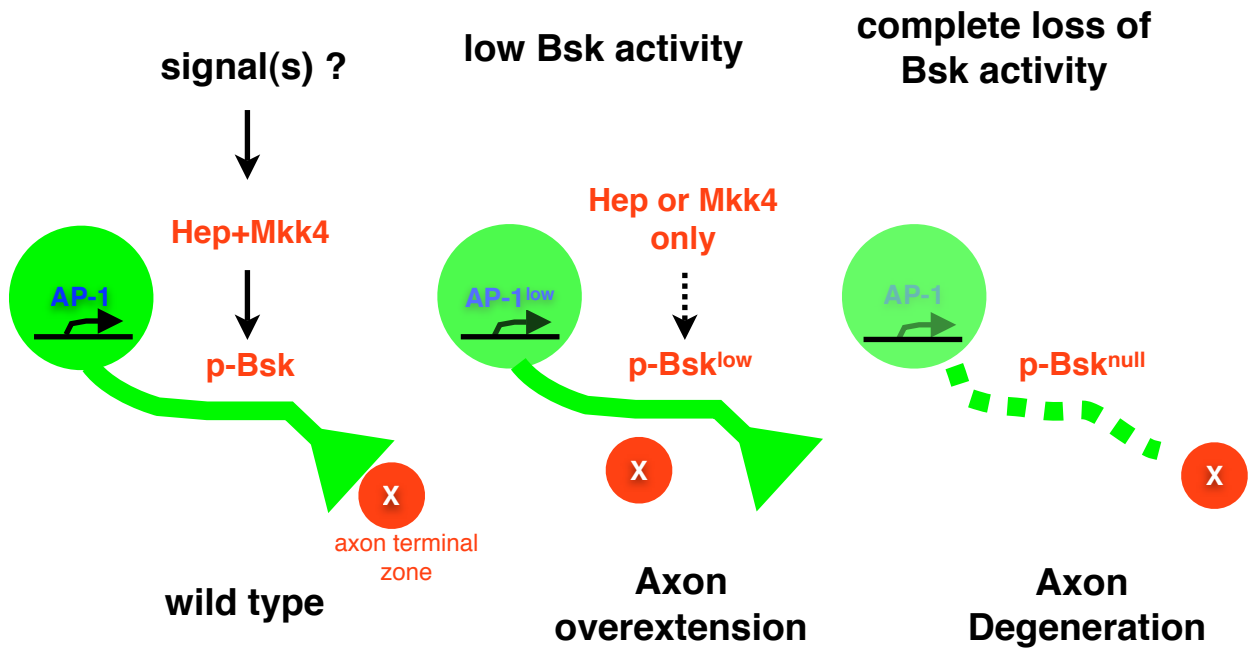

B

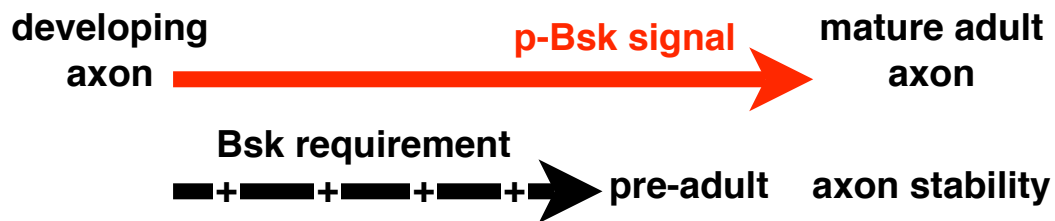

Supplementary  
Figure 9  
Rallis

Supplement: Supplementary Figure 9 — . The role activity level and signal duration plays in Bsk signaling in neurons. A working model of Bsk-dependent axon stability derived from this study. (A) Hep and Mkk4 activities determine the level of Bsk/AP-1 signals in MB neurons resulting in wild-type (normal activity), axon overextension (partial activity), or degenerating (completely loss of Bsk/AP-1) axons. (B) A temporal model of Bsk-dependent axonal morphogenesis. Bsk activity (measured by the P-Bsk signal) is detected throughout development and adulthood (up to 14 days post-eclosion; unpublished observations). Our genetic results show, to maintain optimal axonal stability, Bsk is required throughout the pre-adult, developmental phase. Furthermore, the quantification studies from Bsk TARGET RNAi and rescue analysis show these signals act additively (see Discussion). [file mmc9.pdf]

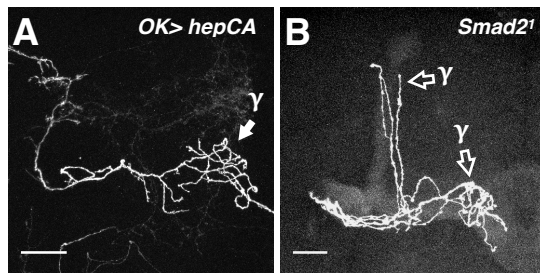

## Supplementary Figure 10 Rallis

Supplement: Supplementary Figure 10 — . Forced expression of constitutively active JNK signals does not block MB axon pruning. (A) Ectopic expression of activated JNKK (HepCA) in γ-neurons (analyzed in single-cell clones) does not result in a block in developmental pruning. (B) For a comparison, loss of Smad signals (Smad2, or Smox-Flybase) results in axon pruning defects, characterized by the presence of larval-stage dorsal and medial γ projections at the adult stage (Zheng et al., 2003). Normal adult γ projections typically have a single medial projection, as seen in bsk-null (Fig. 2J) and HepCA-expressing clones. Scale bar: 20 μm. In both images the cell body sections have been remove to clearly reveal the axonal projections. [file mmc10.pdf]
